# Dietary Supplementation With Various Fat Oils Affect Phytohemagglutinin Skin Test in Broiler Chickens

**DOI:** 10.3389/fimmu.2020.01735

**Published:** 2020-08-14

**Authors:** Hanan Al-Khalaifah, Afaf Al-Nasser

**Affiliations:** Environment and Life Sciences Research Center (ELSRC), Kuwait Institute for Scientific Research, Kuwait City, Kuwait

**Keywords:** broilers, essential oils, immune response, health, intestine, microbial counts, birds, haemocytometric measurements

## Abstract

The current study aimed to investigate the effect of different dietary supplemental oils on the immune status of broilers. One-day-old Cobb 500 broiler chicks were randomly distributed into eight batteries and fed eight experimental diets. There were 680 broilers, 85 birds per battery. The experimental oils were all used at 10% of the total diet. Each dietary treatment (TRT) contained one of the following essential oils: TRT 1 = control group that received a basal diet + soybean oil (SO); TRT 2 = basal diet as in TRT 1 + sunflower oil (SFO); TRT 3 = basal diet as in TRT 1 + canola oil (CO); TRT 4 = basal diet as in TRT 1 + flaxseed oil (FLO); TRT 5 = basal diet as in TRT 1 + fish oil (FO); TRT 6 = basal diet as in TRT 1 + mix of fish oil and soya oil (SO + FO); TRT 7 = basal diet as in TRT 1 + algal biomass oil (DHA); TRT 8 = basal diet as in TRT 1 + echium oil (EO). All samples were taken from 10 birds per treatment (*n* = 10). The immune parameters investigated involved measurement of weights of immune organs as a general indicator, hemocytometric measurements, intestinal microbial count and hindgut acidosis, hindgut volatile fatty acids, and cellular immune response using phytohemagglutinin test. The use of the different dietary treatments did not affect the general health status of the chickens, and the mortality was minimal with no signs of illness or outbreaks. The fact that both the control and the treatment diets were equally consumed would indicate that supplemental oil inclusions did not adversely affect the palatability of the diet by the chickens. At 3 weeks of age, there was no significant effect observed in the microbial counts of the intestine. However, at 5 weeks of age, the highest microbial count was significantly observed for broilers fed EO (7.30%), closely followed by SFO (6.95%), and the least microbial counts were observed for CO (5.63%). No significance was observed for lactic acid bacteria (LAB) and *Salmonella*. There was no significance observed for the effect of the dietary treatments on the hindgut volatile acid in the broilers. Wattle swelling changes were significant between dietary treatments. The results revealed that dietary FLO, FO, and DHA oils induced higher cellular response than the other treatments (*P* = 0.035), representing higher cellular response in these groups. In conclusion, supplemental oils rich in n−3 fatty acids may enhance the immune response in broiler chickens, represented by the intestinal microbial counts and the cellular immune response.

## Introduction

There has been some interest in supplementing poultry meat with functional feed additives to improve poultry production performance. One of these effective feed additives is a fat source. Dietary supplementation with high-fat sources such as oils serves several critical functions in the body. These include providing a source of metabolic energy, acting as critical components of cell membranes and acting as precursors for eicosanoid production (1–7). Examples of oils that were recently used in poultry feed include algal, echium, fish, and linseed oils. Some research studies have reported that oils have positive benefits in broiler production performance, in addition to their ability to modulate lipid profile in meat tissues. However, some inconsistency has been noted in the literature, because some studies have shown the negative effect of high-fat diets on broiler chicken production, whereas other studies show no effect (8–14).

In addition, these oils contain high levels of n−3 polyunsaturated fatty acids (PUFAs) that have recently received considerable attention in both human and animal nutrition. Poultry meat has been enriched with these fatty acids to increase the low human consumption of long chain n−3 PUFAs, which are known to have great nutritional benefits (1, 15–17).

Polyunsaturated fatty acid exerts immunomodulatory effect by affecting intercellular signals that change response of leukocytes as a result of antigenic stimulation. This involves down-regulation or up-regulation of different cytokines such as interleukin 1β (IL-1β), interferon γ, mammary gland factor, IL-1, IL-4, and IL-2 (18–20). In general, it is claimed that n-3 PUFAs have detrimental effects on chicken immune response and its ability to resist infectious diseases (3, 13, 21, 22). However, there is a debate related to this claim because some studies show no effect (23), whereas others show an enhancement of the immune response (24–26).

The current study aimed to investigate the effect of feeding broiler chickens on diets rich in different supplemental oils on the immune response of broiler chickens. The immune investigations involved measurement of weights of immune organs as a general indicator, hemocytometric measurements, intestinal microbial count, and hindgut acidosis, hindgut volatile fatty acids, and cellular immune response using phytohemagglutinin (PHA) test.

## Materials and Methods

### Animals and Diet

One-day-old Cobb 500 broiler chicks were randomly fed on one of eight dietary treatments in eight batteries. Each treatment was replicated five times (17 birds/replicate). The broiler chicks were fed, *ad libitum*, a starter diet from hatch until 7 days of age (1 week), a grower diet from 8 to 21 days of age (2–3 weeks), and a finisher diet from 22 to 35 days of age (4–5 weeks). The diet was corn and/or soy-based that meets the National Research Council. Experimental diets were prepared from basal diets by adding one level (10%) of the supplemental oils. The eight dietary treatments were as follows: TRT 1, which is the control group that received a basal diet with soybean oil (SO); TRT 2, which received the same basal diet as in TRT 1 with sunflower oil replacing the soya oil in the diet (SFO); TRT 3, this group received the same basal diet as in TRT 1 with canola oil replacing soya oil in the diet (CO); TRT 4, this group received the same basal diet as in TRT with flaxseed oil replacing soya oil in the diet (FLO); TRT 5, this group received the same basal diet as in TRT 1 with fish oil replacing soya oil in the diet (FO); TRT 6, this group received the same basal diet as in TRT 1 with a mix of fish oil and soya oil (SO + FO); TRT 7, this group received the same basal diet as in TRT 1 with algal biomass oil replacing soya oil in the diet (DHA); TRT 8, this group received the same basal diet as in TRT 1 with echium oil replacing soya oil in the diet (EO). In all broiler cycles, all samples were taken from 10 birds per treatment (*n* = 10). Table 1 shows the formulation and chemical analysis of the basal broiler feed rations used. Table 2 shows the fatty acid composition of the oils used.

**Table 1:** Chemical analyses of basal broiler diets.

**Ingredient**	**Starter**	**Grower**	**Finisher**
	**(0–7 d)**	**(8–21 d)**	**(22–35 d)**
Corn	55.60	57.60	61.200
Soybean meal	39.400	35.600	32.250
Essential oil^a^	1.350	3.200	3.350
Limestone	1.450	1.450	1.300
Dicalcium phosphate	1.400	1.400	1.200
Salt	0.210	0.210	0.200
l-Lysine	0.120	0.120	0.100
dl	0.270	0.270	0.260
Vitamin–mineral premix^b^	0.200	0.200	0.200
Total %	100	100	100
Nutrient composition			
Chemical analysis			
Crude protein (CP) (%)	24.01	22.36	21.03
Metabolizable energy (kcal/kg)	2932.16	3054.44	3105.30
Fat (g/kg)	3.860	5.748	6.002
Calculated analysis			
Calcium (g/kg)	0.989	0.98	0.87
Phosphorus (g/kg)	0.410	0.402	0.361
Sodium (g/kg)	0.110	0.111	0.108
Lysine (g/kg CP)	1.45	1.34	1.23
Methionine (g/kg CP)	0.665	0.642	0.615
Choline (mg/kg)	1420.73	1329.36	1260.18

a *Each dietary treatment (TRT) contained one of the following essential oils: TRT 1, control group that received a basal diet + soybean oil (SO); TRT 2, basal diet as in TRT 1 + sunflower oil (SFO); TRT 3, basal diet as in TRT 1 + canola oil (CO); TRT 4, basal diet as in TRT 1 + flaxseed oil (FLO); TRT 5, basal diet as in TRT 1 + fish oil (FO); TRT 6, basal diet as in TRT 1 + mix of fish oil and soya oil (SO + FO); TRT 7, basal diet as in TRT 1 + algal biomass oil (DHA); TRT 8, basal diet as in TRT 1 + echium oil (EO)*.

b *Supplied per kg of premix: trans-retinol (A), 12,500,000 IU; cholecalciferol (D_3_), 500,000 IU; α-tocopherol acetate (E), 75,000 mg; thiamine (B_1_), 4,500 mg; riboflavin (B_2_), 8,000 mg; pyridoxine (B_6_), 5,000 mg; vitamin B_12_, 22,000 mg; pantothenic acid, 20,000 mg; folic acid, 2,000 mg; biotin, 200,000 μg; Fe, 100,000 mg; Co,250 mg; Mn, 100 mg; Cu, 10,000 mg; Zn, 80,000 mg; I, 1,000 mg; Se, 300 mg; Mo, 0.5 mg; Ca, 7.7%; P, 0.01%; Na, 0.18%; ash, 97%*.

**Table 2:** Fatty acid composition of the oils used.

**Fatty acids (wt %)**								
	**SO**	**SFO**	**CO**	**FLO**	**FO**	**SO** **+** **FO**	**DHA**	**EO**
C12:0	0.00	0.00	0.00	0.00	0.13	0.12	0.2	0.19
C14:0	0.07	0.08	0.00	0.04	8.67	3.71	0.2	0.18
C14:1	0.00	0.00	0.00	0.00	0.16	0.02	0.04	0.11
C15:0	0.00	0.00	0.00	0.00	0.49	0.22	0.03	0.13
C16:0	10.63	6.88	4.00	5.08	17.15	14.36	12.11	10.21
C16:1n7	0.42	0.11	0.21	0.06	12.66	4.67	1.25	2.55
C17:0	0.39	0.04	0.00	0.06	0.49	0.34	0.21	0.29
C17:1	0.09	0.00	0.00	0.04	1.35	0.53	0.12	0.14
C18:0	4.45	3.39	1.74	3.42	3.59	4.16	0.05	0.07
C18:1n9t	0.00	0.00	0.00	0.00	2.55	0.00	0.03	0.06
C18:1n9c	24.19	24.28	61.57	18.95	7.01	25.58	32.22	10.11
C18:2n6t	1.35	0.00	2.54	0.56	3.51	0.35	0.14	0.91
C18:2n6c	50.09	62.19	18.47	16.50	1.12	30.40	2.52	1.22
C20:0	0.43	0.28	0.59	0.13	0.88	0.11	0.08	0.07
C18:3n6	0.00	0.00	0.20	0.20	0.43	0.14	0.12	0.20
C18:3n3	6.38	0.37	9.19	54.44	0.69	3.59	3.00	14.01
C20:2	0.11	0.04	1.00	0.00	3.18	0.76	0.1	0.09
C22:0	0.00	0.00	0.00	0.03	0.00	0.05	8.99	13.01
C20:3n6	0.47	0.74	0.31	0.12	0.17	0.23	5.50	29.01
C22:1n9	0.00	0.00	0.00	0.00	1.39	0.07	0.91	0.89
C23:0	0.00	0.00	0.00	0.00	2.25	0.06	0.05	0.04
C22:2	0.00	0.03	0.00	0.00	0.60	0.17	0.31	0.98
C24:0	0.00	0.00	0.00	0.00	0.18	0.06	0.00	0.00
C20:5n3	0.00	0.04	0.00	0.00	16.48	4.64	15.5	5.21
C24:1n9	0.16	0.29	0.19	0.08	0.14	0.12	1.99	2.01
C22:6n3	0.15	0.00	0.00	0.02	9.42	2.22	13.87	2.01
Total	99.08	98.45	99.81	99.63	85.11	97.42	99.54	93.7
∑SAT^a^	15.96	10.66	6.33	8.75	33.82	23.22	21.92	28.14
∑MONO^b^	24.86	24.68	61.96	19.12	25.25	31.04	36.56	72.99
∑PUFA^c^	58.56	63.40	31.71	71.85	35.60	43.00	41.06	37.46
∑n−6^d^	51.92	62.92	21.51	17.39	5.23	31.16	8.28	16.03
∑n−3^e^	6.53	0.41	9.19	54.46	26.59	11.08	32.37	21.23
∑n−6:∑n−3^f^	7.95	154.22	2.34	0.32	0.20	2.81	0.30	0.80

*SO, soya oil; SFO, sunflower oil; CO, canola oil; FLO, flaxseed oil; FO, fish oil; SO + FO, soya oil + fish oil; DHA, algal biomass oil; EO, echium oil*.

a *∑SAT = Sum percentage of saturated fatty acids (C12:0, C13:0, C14:0, C15:0, C16:0, C17:0, C18:0, C20:0, C22:0, C21:0, C23:0, C24:0)*.

b *∑MONO = sum percentage of monounsaturated fatty acids (C14:1, C15:1, C16:1, C17:1, C18:1n9trans, C18:1n9cis, C20:1n9, C22:1n9, C24:1n9)*.

c *∑PUFA = sum percentage of polyunsaturated fatty acids (C18:2n−6t, C18:2n−6c, C18:3n−6, C18:3n−3, C20:2n−6, C20:3n−6, C20:4n−6, C20:5n−3, C22:2, C22:5n−3, C22:6n−3)*.

d *∑n−6 = sum percentage of n-6 polyunsaturated fatty acids (C18:2n−6t, C18:2n−6c, C18:3n−6, C20:3n−6, C20:4n−6)*.

e *∑n−3 = sum percentage of n-3 polyunsaturated fatty acids (C18:3n−3, C20:3n−3, C20:5n−3, C20:5n−3, C22:6n−3)*.

f *∑n−6:∑n−3 = ratio of ∑n−6 to ∑n−3*.

### Collection of Samples

The committee of the Poultry Production Department approved the sampling procedure based on official decrees of the Ministry of Agriculture in Egypt (decree no. 27 (1967). Blood samples were collected on weeks 3 and 5 of the broiler cycle in heparinized tubes. Birds were sacrificed by stunning and bleeding. The immune organs (i.e., spleen, bursa of Fabricius, thymus) were surgically removed. The bursa is a discrete organ and can readily be removed; however, thymus of chicken comprised several lobes, and by careful ventral abdominal dissection, under aseptic conditions, maximum thymic tissues were removed and then weighed.

### Hemocytometric Analyses

This involved the evaluation and numeration of immune and red blood cells (RBCs) using a computerized hemocytometer. Blood samples were collected from the branchial vein in vacutainer tubes (K2EDTA) from 10 chickens from each treatment. The samples were instantly analyzed by the Cell-Dyn 3500 hematology system (Abbott Laboratories, Abbott Park, IL, USA) to measure the total and differential white blood cells and blood quality parameters including RBCs, hemoglobin (HGB), hematocrit, mean corpuscular volume, mean corpuscular HGB, mean corpuscular HGB concentration, red cell distribution width, and platelet count.

### Intestinal Microbial Count and Hindgut Acidosis

The microbial flora and the hindgut acidosis of the broilers were assessed as described by Al-Khalaifa, Al-Nasser (27) as follows:

Microbial Counts: The chickens were first disinfected. The abdominal area was cleared of all feathers, and the area was sterilized with 70% ethanol prior to dissection. An incision was made removing all skin so as to reach the digestive system. This was followed by exposure of lower intestine; the ceca were then removed and weighed. For microbial analysis, the cecal content was extracted; each cecum was squeezed onto a sterile Petri dish, after which the ceca were vertically and rinsed with 0.85% (wt/vol) NaCl sterile solution (1:9 vol/vol) to remove any debris. The cecal extract was then placed into a stomacher bag and homogenized for 3 min and was then used for microbial analysis. Lactic acid bacteria, *Escherichia coli*, and *Salmonella* counts were determined using standard microbiological methods as described by Lorch (28), and samples were analyzed by applying the spreading technology. *Escherichia coli* and *Salmonella* count experiments were conducted using Brilliance *E. coli* selective and Xylose–Lysine–Deoxycholate agar media (Oxoid), respectively, whereas lactic acid bacteria (LAB) experiments were conducted using de Man–Rogosa–Sharpe media (Oxoid). For the analysis, serial dilution was made from the crude samples. Approximately 0.1 mL of the prepared sample was spread onto the surface of the media with a sterile spreader. The plates were incubated aerobically for 24 h at 37°C for both *E. coli* and *Salmonella*, whereas for LAB, the plates were incubated anaerobically for 48 h at 37°C. The colonies were counted at the end of the incubation periods and were transformed to log values.
Hindgut Acidosis: This is carried out by lowering the pH in the cecum or colon. The tissues were removed from duodenum to the ileum and ceca. The hindgut is the part of the intestine from the yolk sac diverticulum to the ileocecal junction; the hindgut digesta were collected into tubes, a pH probe was placed into the digesta, and the pH was recorded.

### Hindgut Short Volatile Fatty Acids

This test was done at weeks 3 and 5 in each broiler cycle. Ten chickens from each treatment were used. Samples used were intestinal fluid taken from the chickens. Approximately 1.50 g of thawed digesta was diluted with distilled water (1:1 wt/vol) in a screw-capped tube. After homogenization and centrifugation, 1 mL of clear supernatant was used to analyze the volatile fatty acid profile using gas chromatography (GC) techniques as described by Al-Khalaifa, Al-Nasser (27). The separation fatty acids using GC is that they differ in temperature at which they become volatile. This is dependent on the number of carbons and position of double bonds in the molecules. The injection temperature was set at 140°C, and helium was used as the carrier gas with a precolumn split ratio of 50:1 and a head pressure of 37.34 psi. The temperature program recommended for the optimal separation of methyl esters is as follows:

1. The oven was initially heated to 100°C and held for 1 min, and the temperature was then increased to 240°C at 4°C per minute.
2. The fatty acid methyl ester (FAME) was diluted in 100 μL of hexane, 1 μL of which was injected onto the column using an Agilent 7,683 series autosampler.
3. The FAME was identified by comparing the retention time against a known standard (Supelco Volatile Free Acid Mix; Sigma-Aldrich, Laramie, Wyoming, USA). FAMEs were detected using a flame ionization detector.
4. The chromatograms were plotted and analyzed using the HP Chemstation software package.

The GC machine (Agilent Technologies GC system, Wilmington, DE, USA) was standardized using the short-chain volatile fatty acid standard mixes. All of the required fatty acid peaks were obtained and standardized. The machine was ready to analyze the samples.

### Cellular Immune Response Using PHA Test

The cellular immune response was investigated in broiler chickens using PHA skin test. This test involved subcutaneous injection of a mitogen (PHA) and measurement of subsequent swelling as a surrogate of T cell–mediated immunocompetence. The PHA was dissolved in cell-culture grade/pyrogen-free phosphate-buffered saline (PBS), and the resulting concomitant swelling was quantified at the site of injection over time. The resulting swelling, usually measured 24 h after injection, was interpreted as an index of cell-mediated Immunocompetence (29). At 5 weeks of age, 10 chickens per treatment were injected with PHA. The injection site (wattle) was marked prior to injection, and the thickness of the injection site was measured by micrometer. After then, the birds were injected intradermally in the wattle with 0.5 mg of PHA-P in 0.1 mL of PBS. Post-injection thickness was typically measured at 24 h after injection, yet 24 h did not reflect the peak of the reaction; it could be measured (to nearest 0.01 mm) at 0, 24, 48, and 72 h after PHA-P injection. Wattle swelling was calculated as the difference between the thickness of the wattle prior to and after injection of PHA-P.

### Statistical Analysis

The overall differences between dietary treatments were analyzed using one-way analysis of variance, and the general linear model procedure of Minitab was applied. Differences between the treatment groups were considered statistically different at *P* ≤ 0.05. When significant differences occurred (*P* ≤ 0.05), treatment mean differences were identified by pairwise comparison using Bonferroni test.

## Results

All broilers used in the current study appeared healthy, and no significant mortality occurred throughout the experimental period. The fact that both the control and the treatment diets were equally consumed would indicate that supplemental oil inclusions did not adversely affect the palatability of the diet by the chickens.

### Measurement of Weights of Immune Organs

The effect of different supplemental oils on tissue weight in chickens of broiler cycle 1 is shown in Table 3. Values are presented as a percentage of body weight to account for differences in weights between different birds. Results showed that feeding broiler chickens on diets rich in the different supplemental oils did not significantly affect the weights of the immune organs of broiler chickens.

**Table 3:** Effect of different supplemental oils on tissue weight in broiler chickens.

**Diet**	**Abdominal fat**	**Heart**	**Spleen**	**Liver**	**Bursa**	**Thymus**	**Breast**	**Leg + thigh**
**g/kg**		**% of Body weight**						
SO	0.84	0.52	0.12	3.14	0.13	0.43	6.24	3.70
SFO	0.91	0.53	0.08	2.11	0.21	0.54	5.11	4.63
FLO	0.75	0.46	0.09	2.15	0.12	0.43	5.40	3.19
FO	1.15	0.53	0.11	2.44	0.15	0.35	7.27	3.30
CO	1.41	0.49	0.10	1.74	0.18	0.45	4.51	3.17
SO + FO	0.87	0.46	0.12	2.45	0.26	0.46	5.26	3.67
DHA	1.18	0.53	0.14	3.45	0.25	0.92	4.77	3.18
EO	1.64	0.21	0.10	2.30	0.19	0.43	4.92	4.11
SE Mean mmMean	0.31	0.24	0.02	0.42	0.05	0.26	0.59	0.56
*P*-value	0.480	0.412	0.327	0.158	0.459	0.832	0.078	0.534

*SO, soya bean oil; SFO, sunflower oil; FLO, flax seed oil; FO, fish oil; CO, canola oil; SO + FO, soybean oil + fish oil; DHA, algal biomass; EO, echium oil*.

*Differences between the treatment groups are statistically different at P ≤ 0.05, n = 10*.

### Hemocytometric Measurements

Table 4 shows the hematological and biochemical parameters of 5-week-old broiler different dietary supplemental oils. Results in Table 3 shows that there is no significant effect of the different supplemental oils on the hematological and biochemical characteristics of the blood.

**Table 4:** Hematological and biochemical parameters of 5-week-old broiler fed different dietary supplemental oils.

**Variables**	**SO**	**SFO**	**FLO**	**FO**	**CO**	**FO + SO**	**DHA**	**EO**	**SEM**	***P***
WBC	55.50	62.20	35.20	49.20	40.31	30.34	25.51	20.38	0.515	0.120
Neutrophils	21.01	15.95	25.21	20.94	19.98	15.98	20.45	21.20	0.500	0.230
Lymphocytes	40.97	41.20	42.98	42.97	35.21	39.84	40.02	42.94	0.420	0.450
Monocytes	30.21	22.97	19.04	22.97	34.00	30.74	35.94	32.00	0.394	0.150
Eosinophils	0.50	0.15	0.14	0.08	0.06	0.05	0.18	0.31	0.310	0.161
Basophils	18.33	17.99	10.98	19.20	18.00	20.21	23.10	20.10	0.513	0.941
RBC	2.51	3.10	2.54	2.01	3.00	2.50	4.60	6.01	0.380	0.522
HGB	15.12	16.20	13.98	14.00	13.98	12.99	13.21	16.98	0.470	0.321
HCT	41.01	35.21	32.01	26.21	35.97	35.10	25.94	24.10	0.597	0.850
MCV	150.98	142.55	140.00	123.50	141.00	154.20	140.01	151.20	0.410	0.231
MCH	50.21	55.32	59.21	45.68	49.87	51.97	50.78	59.31	0.395	0.384
MCHC	30.21	34.65	36.00	29.31	30.89	31.54	32.41	33.54	0.740	0.201
RDW	12.52	13.54	15.21	10.55	12.97	9.87	15.54	11.97	0.551	0.303
PLT	29.87	19.54	12.01	9.21	6.01	6.12	8.55	6.30	0.300	0.197

*WBC, white blood cell; RBC, red blood cell; HGB, hemoglobin; HCT, hematocrit; MCV, mean corpuscular volume; MCH, mean corpuscular hemoglobin; MCHC, mean corpuscular hemoglobin concentration; RDW, red blood cell distribution width; PLT, platelet count; SO, soya bean oil; SFO, sunflower oil; FLO, flax seed oil; FO, fish oil; CO, canola oil; SO + FO, soybean oil + fish oil; DHA, algal biomass; EO, echium oil; SEM, standard error mean. Differences between the treatment groups are statistically different at P ≤ 0.05, n = 10*.

### Intestinal Microbial Count and Hindgut Acidosis

Table 5 shows the effect of different dietary treatments on the microbial count and hindgut acidosis in 3-week-old broiler chickens. There was no significance observed on the microbial counts of the 3-week-old broiler chickens (Table 5).

**Table 5:** Effect of different dietary treatments on microbial count in 3-week-old broiler chickens.

		**Log LAB**	**Log *E. coli***	**Log *Salmonella***	**pH**
Treatments	SO	6.41	0	0	6.59
	SFO	6.14	0	0	7.01
	FLO	5.38	0	0	6.98
	FO	5.75	0	0	7
	CO	6.95	2.5	0	6.54
	FO + SO	7.2	0	0	6.99
	DHA	6.85	0	0	7.23
	EO	6.63	0	0.05	6.84
	SEM	0.381	0.883	0.431	0.255
	*P*-value	0.097	0.493	0.515	0.596

*SO, soya oil; SFO, sunflower oil; FLO, flaxseed oil; FO, fish oil; CO, canola oil; FO + SO, fish + soya oil; DHA, docosahexaenoic acid; EO, echium oil; SEM, standard error mean. Differences between the treatment groups are statistically different at P ≤ 0.05, n = 10*.

The effect of the different dietary treatments on the microbial count and hindgut acidosis in 5-week- old broiler chickens is shown in Table 6. Results in Table 6 revealed that the effect of dietary treatments on 5-week-old broiler chickens show significance (*P* ≤ 0.05) for the *E. coli* microbial counts. The highest microbial count was observed for broilers fed EO (7.30%), closely followed by SFO (6.95%), and the least microbial counts were observed for CO (5.63%). No significance was observed for LAB and *Salmonella*.

**Table 6:** Effect of different dietary treatments on microbial count in 5-week-old broiler chickens.

		**Log LAB**	**Log *E. coli***	**Log *Salmonella***	**pH**
Treatments	SO	7.62	6.11^ab^	0	7.35
	SFO	8.3	6.95^a^	0	7.12
	FLO	8.6	6.72^ab^	1.15	6.99
	FO	7.74	6.17^ab^	0	6.25
	CO	7.41	5.63^b^	0	7.55
	FO + SO	7.36	6.58^ab^	0	6.78
	DHA	7.71	6.37^ab^	0	7.03
	EO	8.06	7.30^a^	1	6.54
	SEM	0.316	0.216	0.538	0.345
	*P*-value	0.196	0.012	0.573	0.631

*SO, soya oil; SFO, sunflower oil; FLO, flaxseed oil; FO, fish oil; CO, canola oil; FO + SO, fish + soya oil; DHA, docosahexaenoic acid; EO, echium oil; SEM, standard error mean. Differences between the treatment groups are statistically different at P ≤ 0.05, n = 10*.

a, b *Indicate significant differences between means*.

### Hindgut Short Volatile Fatty Acids

The results on the effect of different dietary essential oils on the short-chain fatty acids of broilers are shown in Table 7. There was no significance observed for the effect of these dietary treatments on the volatile acid composition in the broilers.

**Table 7:** Short fatty composition of 5-week-old broilers fed different dietary treatments.

	**Fatty acids**	**Acetic acid**	**Propionic acid**	**Butyric acid**	**Isovaleric acid**	**Valeric acid**
Treatments	SO	60.25	0.02	0	0.98	0.08
	SFO	79.87	0.09	0	1.31	0.65
	FLO	99.65	0	1.01	1.5	1.12
	FO	50.87	0.012	0.98	0.08	0.88
	CO	101	0.054	0.052	0.09	0.51
	FO + SO	86.54	0	1.03	1.03	0.61
	DHA	90.54	0.08	0.04	0.19	0.05
	EO	85.21	0.055	0	0.97	0.54
	SEM	0.235	0.312	0.298	0.512	0.443
	*P*-value	0.548	0.213	0.345	0.124	0.2

*SO, soya oil; SFO, sunflower oil; FLO, flaxseed oil; FO, Fish oil; CO, canola oil; FO + SO, fish + soya oil; DHA, docosahexaenoic acid; EO, echium oil; SEM, standard error mean. Differences between the treatment groups are statistically different at P ≤ 0.05, n = 10*.

### Cellular Immune Response Using PHA Test

Wattle swelling changes as affected by the different dietary treatments are shown in Table 8. The results revealed that dietary FLO, FO, and DHA oils induced higher cellular response than the other treatments (*P* = 0.035).

**Table 8:** Wattle swelling changes as affected by the different dietary treatments.

	**Wattle swelling (mm)**	
Treatments	SO	1.06^b^
	SFO	1.25^b^
	FLO	2.15^a^
	FO	2.24^a^
	CO	1.13^b^
	FO + SO	1.57^b^
	DHA	2.42^a^
	EO	1.09^b^
	SEM	0.256
	*P*-value	0.035

a, b *means within rows with no common superscripts are significantly different. SO, soya oil; SFO, sunflower oil; FLO, flaxseed oil; FO, fish oil; CO, canola oil; FO + SO, fish + soya oil; DHA, docosahexaenoic acid; and EO, echium oil; SEM, standard error mean. Differences between the treatment groups are statistically different at P ≤ 0.05, n = 10*.

## Discussion

### Measurement of Weights of Immune Organs

Conversely, it was reported in some studies that feeding PUFAs to chickens (30) and mice (31) results in increased spleen weights. Assessment of weights and cellularity of immune organs has been traditionally used to initially estimate the general immune status in birds. The main immune organs in poultry are the thymus, spleen, and bursa of Fabricius. The relative weights of immune organs reflect the development and function of the immune system inclusive of the humoral and cellular immunity. Consequently, the mass and cellular status of these organs can generally indicate an immune competence (22, 32, 33).

### Hemocytometric Measurements

It is noteworthy that supplementary oil inclusions in the current study did not cause anemia for the chickens, as there was no significant effect on the RBC counts and HGB concentration. Differences in the counts of some immune cells as a result of different supplementary oils fall within the normal expected ranges, and no serious mortality rates have been observed. Investigating the effect of immunomodulators on hematology and blood quality had been used to define clinical pathology and disease diagnosis, as hematological analysis includes studying cellular and fluid portions of blood and the circulating white blood cells, which constitute the defense elements against pathogens (34). In addition, hematology and blood quality analysis are usually used to assess the health and physiological status of human and animals. Any changes in hematological parameters or blood quality may indicate immunomodulation caused by certain factors (35).

### Intestinal Microbial Count and Hindgut Acidosis

Our study is in line with that of Toghyani, Girish (36). The authors conducted a pair-feeding study to determine if reduced feed intake in broilers fed high canola meal (CM) diets affected growth performance and can increase dietary amino acid. Five experimental wheat-based diets were formulated as follows: soybean meal (SBM) diet, high CM diet with normal AA concentration, and high CM diets with 3, 6, or 9% additional AA concentration (Lys, Met + Cys, Thr, Ile, Arg, and Val). Another group of birds was pair-fed with SBM diet to the consumption levels of birds fed CM diet with normal AA. Their results indicated that composition of microbiota in the ceca was not affected by the treatments.

Poorghasemi, Seidavi (37) conducted a study to determine the effect of dietary fat source in the feed of broiler chickens with completely random design with five treatments on Ross 308 broilers. The first treatment included 4% animal fat of tallow, the second treatment with 4% fat plant of canola oil, the third treatment with 4% plant fat of sunflower oil, the fourth treatment with 2% animal fat of tallow +2% plant fat of canola oil, and the fifth treatment with 2% animal fat of tallow and 2% plant fat of sunflower oil. The results of their study showed that adding plant and animal fat had no statistically significant effect on total microbial population and LAB counts (*P* > 0.05), but addition of fat sources significantly affected Lactobacillus bacteria (*P* ≤ 0.05). There was no significant difference between the hindgut acidosis, represented by the short-chain volatile fatty acids, of the different dietary groups.

### Hindgut Short Volatile Fatty Acids

In another similar study, 294 1-day-old Cobb broiler chickens were fed six dietary treatments consisting of basal diets and four strains of the probiotic *Lactobacillus*. The results showed that no significance was observed for growth performance, as well as no effect was seen on pH and concentrations of short-chain fatty acids (38).

### Cellular Immune Response Using PHA Test

The PHA test has been used after the pioneering work by Goto, Kodama (29), who showed a reduction of the skin response in thymectomized chickens (thus being unable to produce circulating T cells). Histologically, this reaction was characterized by a perivascular accumulation of lymphocytes and macrophage migration in the central layer of the wattles. Heterophilic infiltration was observed mostly at early hours and waned thereafter. These responses were significantly decreased in cases of neonatal thymectomy. Therefore, the PHA skin test was considered to be a thymus-dependent response (39). Phytohemagglutinin possesses the potential to crosslink T-cell surface receptors and trigger proliferation of T cells without the requirement of antigen-presenting cells. Injecting PHA into wing web or wattle or subcutaneously into skin tissues and then measuring swelling response prior to and after injection so as to measure immune index especially adaptive immunity is mostly used in nutritional immunology studies. Although PHA is a T-cell mitogen that stimulates proliferation of T cells, it also can stimulate a number of other immune cells. When injected subcutaneously, it initiates complex reactions such as infiltration by subsets of immune cells. Phytohemagglutinin also induces a local inflammatory response to enhance blood flow and cellular infiltration at the site of injection. Hence, at 24 or 48 h of PHA injection, the ability of PHA to agglutinate RBCs instead of T cells is reason for increase in skin swelling at the site of injection. Phytohemagglutinin swelling is a dynamic response inclusive of cells from innate and acquired immunity and thus cannot be solely relied upon as representation of T-cell proliferation (40). Thus, our study showed that supplementing omega-3 or omega-6 PUFAs in broilers significantly increased the PHA web response, generally considered an *in situ* parameter of initial inflammation followed by T-cell proliferation, which is indicative of the T cell–mediated immune response.

To summarize, the findings of our study showed no significance for tissue weight of immune organs, hematological and biochemical parameters, and microbial counts of 3-week-old broilers except for *E. coli* microbial counts in 5-week-old broilers fed EO. No significance was also observed for hindgut short-chain volatile fatty acids. However, results revealed that dietary FLO, FO, and DHA oils induced higher cellular response than the other treatments for wattle swelling by the PHA test. A study by Al-Khalifa, Givens (22) on incorporating different levels (0, 30, 50, or 60 g/kg) of FO in the diets of 3-week-old broilers showed FO did not have any effect on spleen but increased thymus and bursal weights at 50 g/kg. There was no significant effect of FO on immune cell phenotypes in the spleen, thymus, bursa, or blood. Feeding 60 g/kg of FO significantly decreased the percentage of monocytes engaged in phagocytosis. Dietary fatty acids may modulate the broiler chicken performance and immune response in a dose-dependent manner. Phytohemagglutinin testing is widely used in poultry studies to examine cellular immunocompetence as it is fairly simple and cost-effective. Injecting PHA results in innate immune response followed by a secondary response reflective of polyclonal cell-mediated immunity. The greater the swelling response, the greater is the cellular immune responsiveness. However, it still uncertain whether a larger swelling is indicative of a better and balanced immune response, but low PHA response is related to immunosuppression (41).

## Conclusion

The trend of using PUFAs particularly in human and animal nutrition has gained a lot of attention in the recent years. Although many studies indicate a positive response to these fatty acids, there arise every now and then some studies wherein the PUFAs could have a detrimental effect or no effect altogether. In conclusion, the studied level of oils was not able to improve immune response of the broilers but only showed a significance for PHA web response. This could be due to a number of factors, one of which could be the level of oils used; perhaps increasing the levels of supplemental oils would have yielded different results. Further research is essential so as to analyze all factors contributing to interactions of dietary fat and broiler immunity and to allow dietary fat manipulation to optimize broiler immunity.

## Data Availability Statement

The original contributions presented in the study are included in the article/supplementary material, further inquiries can be directed to the corresponding author/s.

## Ethics Statement

The animal study was reviewed and approved by the committee of the Poultry Production Department who approved the sampling procedure based on official decrees of the Ministry of Agriculture in Egypt (Decree No. 27, 1967).

## Author Contributions

Both authors listed have made a substantial, direct and intellectual contribution to the work, and approved it for publication.

## Conflict of Interest

The authors declare that the research was conducted in the absence of any commercial or financial relationships that could be construed as a potential conflict of interest.
